# Impact of prosthesis-patient mismatch on early and late mortality after aortic valve replacement

**DOI:** 10.1186/1749-8090-8-96

**Published:** 2013-04-17

**Authors:** Bart M Koene, Mohamed A Soliman Hamad, Wobbe Bouma, Massimo A Mariani, Kathinka C Peels, Jan-Melle van Dantzig, Albert H van Straten

**Affiliations:** 1 Department of Cardiothoracic Surgery, Catharina Hospital, Eindhoven, The Netherlands; 2 Department of Cardiothoracic Surgery, University Medical Center Groningen, Groningen, The Netherlands; 3 Department of Cardiology, Catharina Hospital, Eindhoven, The Netherlands

**Keywords:** Prosthesis, Heart valves, Aortic valve, Replacement, Prosthesis-patient mismatch, Statistics, Survival analysis

## Abstract

**Background:**

The influence of prosthesis-patient mismatch (PPM) on survival after aortic valve replacement (AVR) remains controversial. In this study, we sought to determine the effect of PPM on early (≤30 days) and late mortality (>30 days) after AVR or AVR combined with coronary artery bypass grafting (AVR with CABG).

**Methods:**

Between January 1998 and March 2012, 2976 patients underwent AVR (n= 1718) or AVR with CABG (n=1258) at a single institution. PPM was defined as an indexed effective orifice area (EOAI) ≤0.85 cm^2^/m^2^ and patients were divided into two groups based on the existence of PPM. Cumulative probability values of survival were estimated with Kaplan-Meier method and compared between groups using Breslow test. Univariate and multivariate independent predictors of early mortality were identified using logistic regression. Cox proportional-hazard regression analysis was used to determine univariate and multivariate independent predictors of late mortality.

**Results:**

Early mortality was 6.7% in the PPM group vs 4.7% in the group with no PPM (p=0.013). Late mortality for the PPM group at 1, 5 and 10 years was 4%, 16% and 43%, respectively. Late mortality for the group with no PPM at 1, 5 and 10 years was 4%, 15% and 33% respectively. Independent predictors of early mortality included age, severely impaired left ventricular (LV) function, endocarditis, renal dysfunction, chronic obstructive pulmonary disease (COPD) and cardiopulmonary bypass (CPB) time. Multivariate independent predictors of late mortality included age, severely impaired LV function, diabetes, peripheral vascular disease (PVD), renal dysfunction, history of a cerebrovascular accident (CVA), CPB time and a history of previous cardiac surgery. PPM was not an independent predictor of early or late mortality.

**Conclusion:**

PPM is not an independent predictor of both early and late mortality after AVR or AVR combined with CABG.

## Background

Prosthesis-patient mismatch (PPM) after aortic valve replacement (AVR) occurs when the effective orifice area (EOA) of the implanted valve prosthesis is too small compared to the body surface area (BSA) of the patient [1]. PPM is expressed by the indexed EOA (EOAI). The EOAI is calculated by dividing the corresponding EOA of each valve type and size by each patient’s BSA [1,2].

Several studies have shown that PPM is associated with increased early or late mortality after AVR [3-6]. Other studies contradict these findings and report that PPM does not have a significant impact on survival [7-14]. Comparison of results is hampered by differences in cut-off values for EAOI. Cut-off values in literature range from <0.6 to <1.1 cm^2^/m^2^[15]. Comparison of results is also hampered by the fact that some authors use the published normal *in vitro* EOA values provided by the manufacturers to identify PPM [2,14,16], while others use the published normal *in vivo* EOA values to identify PPM [2,5,17].

In this study, we sought to determine the effect of PPM (cut-off value EOAI ≤0.85 cm^2^/m^2^ as described by Pibarot and colleagues [1]) on early (≤30 days) and late (>30 days) mortality after AVR or aortic valve replacement combined with coronary artery bypass grafting (AVR with CABG).

## Methods

### Study design

This is a retrospective observational study on consecutive patients. Data were obtained from the institutional database, normally utilized for patient care. Clinical data, echocardiographic data, catheterization data and surgical reports were entered into the institutional database prospectively and analyzed retrospectively. Because standard clinical follow-up check-ups were used and data were collected and analyzed anonymously, the need for informed consent was waived by the Medical Ethical Committee.

### Patients

Between January 1998 and March 2012, 2976 patients (mean age 68±11 years) underwent AVR (n=1718) or AVR with CABG (n=1258) at a single institution using a mechanical or a stented biological valve prosthesis. Patients who underwent AVR with implantation of a stentless biological valve were not considered. No aortic annular enlargement procedures were performed.

Follow-up data concerning mortality were gathered using the databases of health insurance companies. The remaining data that could not be retrieved from these databases were obtained by contacting patients’ general practitioners and if necessary by contacting local government authorities. Nineteen patients were lost to follow-up; mean follow-up was 5.7±3.7 years (range 0.0-14.7 years). Patients lost to follow-up were excluded from our analysis. Late mortality was defined as death occurring later than 30 days after surgery.

Based on the EOAI the patients were split into two groups. Patients with an EOAI ≤0.85 cm^2^/m^2^ were assigned to the group with PPM and patients with an EOAI >0.85 cm^2^/m^2^ to the group without PPM.

### Surgical technique

All patients underwent surgery using a standard technique. After a median sternotomy the ascending aorta and right atrium were cannulated and normothermic extracorporeal circulation with non-pulsatile flow was instituted. Myocardial protection was obtained using cold crystalloid cardioplegia (St. Thomas solution) or warm blood cardioplegia. According to surgeon’s preference, cardioplegia was administered in an antegrade fashion through the aortic root and/or selectively in both coronary ostia to induce and maintain cardiac arrest. Retrograde administration of cardioplegia was not used. Concomitant myocardial revascularization was performed in 1258 patients. The distal anastomoses were performed before AVR. An overview of implanted prosthetic valve types is shown in Table 1. No aortic annulus enlargement techniques were used.

**Table 1:** Prosthetic valve distribution (n=2957)

**Variable**	**Value**
Prosthetic valve diameter (mm)	
Median	23 mm
Prosthetic valve type	
Mechanical	1476 (49.9)
St. Jude Medical Standard	899 (30.4)
ATS	442 (14.9)
St. Jude Medical Regent	93 (3.1)
St. Jude Medical HP	42 (1.4)
Biological (stented)	1487 (50.1)
Carpentier-Edwards Magna	146 (4.9)
Carpentier-Edwards Perimount	611 (20.7)
Sorin Mitroflow	325 (11.0)
Medtronic Mosaic	134 (4.5.)
St. Jude Medical Epic	176 (6.0)

Data are number of patients (%).

### Prosthesis-patient mismatch (PPM)

PPM was expressed by the EOAI. The EOAI was calculated by dividing the corresponding EOA of each valve type and size (registered *in vitro* values published by each manufacturer) by each patient’s BSA [1,2]. PPM was defined as EOAI ≤0.85 cm^2^/m^2^[1]. Subgroup analysis of moderate PPM (EOAI >0.65 cm^2^/m^2^ and ≤0.85 cm^2^/m^2^) (n=330) and severe PPM (EOAI ≤0.65 cm^2^/m^2^) was not performed because of the small group size of patients with severe PPM (n=14).

### Statistics

Continuous variables were expressed as mean ± SD. Categorical variables were expressed as percentages. Mean values were compared by using independent-samples T-test or its non-parametric alternative, the Mann–Whitney U test, for continuous variables and Pearson’s Chi-squared test for categorical variables.

Cumulative probability values of survival were estimated with Kaplan-Meier method and compared between groups by using Breslow test.

Logistic regression was used to identify univariate predictors and multivariate independent predictors of early mortality. Odds ratios (OR) were reported with 95% confidence intervals (CI). Goodness of fit of the final model was assessed with the Hosmer-Lemeshow goodness-of-fit test.

Cox proportional-hazard regression analysis was used to determine univariate predictors and multivariate independent predictors of late mortality. Hazard ratios (HR) were reported with 95% confidence intervals (CI).

Variables considered as potential predictors for multivariable modeling were selected by univariate analyses (p<0.05) and were subsequently selected by stepwise forward selection, with entry and retention in the model set at a significance level of 0.05. Goodness of fit of the final model was assessed with the Chi-squared goodness-of-fit test.

All calculations were performed using a commercially available statistical package (SPSS 19.0; SPSS Inc., Chicago, IL). Statistically significant differences were established at p<0.05.

## Results

### Characteristics of the patient population

After excluding 19 patients lost to follow-up, 2957 patients were analyzed. 1701 patients underwent AVR and 1256 patients underwent AVR with CABG. 344 patients had an EOAI ≤0.85 cm^2^/m^2^ and were allocated to the PPM group. The remaining 2613 patients with an EOAI >0.85 cm^2^/m^2^ were allocated to the group without PPM. Patient characteristics are shown in Table 2. The PPM group had significantly more female patients (47.4 vs 36.9%, p<0.001). The patients in the PPM group had a significantly higher average body weight (84±15 kg vs 77±13 kg, p<0.001), a higher average body mass index (BMI) (29.3±5.0 vs 26.8±4.0 kg/m^2^, p<0.001), a higher average BSA (1.95±0.18 vs 1.89±0.19 m^2^, p<0.001), but there was no significant difference in average height. In the PPM group significantly more mechanical valve prostheses were implanted (69.8% vs 47.3%, p<0.001), the average cardiopulmonary bypass (CPB) time was longer (99±43 vs 93±37 min, p=0.022) and significantly more patients had had previous cardiac surgery (17.2% vs 8.6%, p<0.001). With regard to the distribution of comorbidities between the two groups, only the prevalence of diabetes was significantly higher in the PPM group (27.9% vs 15.8%, p<0.001).

**Table 2:** Preoperative, perioperative and postoperative data

**Variable**		**No PPM**	**PPM**	***P***
		**(EOAI > 0.85 cm**^**2**^**/m**^**2**^**) n= 2613**	**(EOAI ≤0.85 cm**^**2**^**/m**^**2**^**) n = 344**	
Age, years		68±11	68±11	0.557
Sex				
Female		965 (36.9)	163 (47.4)	<0.001
Endocarditis		120 (4.6)	15 (4.4)	0.846
Preoperative LV function				
Severely impaired	(EF <30%)	92 (3.5)	11 (3.2)	0.759
Hypertension		1125 (43.1)	166 (48.3)	0.067
Diabetes Mellitus		413 (15.8)	96 (27.9)	<0.001
Body weight, kg		77±13	84±15	<0.001
Height, cm		170±9	170±9	0.807
Body surface area (BSA), m^2^		1.89±0.19	1.95±0.18	<0.001
Body mass index (BMI), kg/m^2^		26.8±4.0	29.3±5.0	<0.001
Peripheral vascular disease (PVD)		261 (10.0)	41 (11.9)	0.266
Renal dysfunction		147 (5.6)	19 (5.5)	0.938
Chronic obstructive pulmonary disease (COPD)		462 (17.7)	72 (20.9)	0.141
Cerebrovascular accident (CVA)		122 (4.7)	236 (6.7)	0.103
Prosthetic valve diameter, mm				
Median		23	23	
Prosthetic valve type				
Mechanical		1236 (47.3)	240 (69.8)	<0.001
Concomitant coronary artery bypass grafting (CABG)		1121 (42.9)	135 (39.2)	0.197
Cardiopulmonary bypass (CPB) time, min		93±37	99±43	0.022
Aortic cross-clamp time, min		68±26	71±30	0.077
Effective orifice area (EOA), cm^2^		2.14±0.44	1.54±0.17	<0.001
Indexed effective orifice area (EOAI), cm^2^/m^2^		1.14±0.21	0.79±0.06	<0.001
Additive EuroSCORE		6±3	6±3	0.095
Logistic EuroSCORE		7.66± 8.14	8.64±9.29	0.146
Previous cardiac surgery		226 (8.6)	59 (17.2)	<0.001
Mean follow-up, years		5.6±3.7	6.3±4.0	<0.001
Mortality ≤30 days		100 (3.8)	23 (6.7)	0.013

Data are mean ± SD or percentage of patients.

### Early mortality

Early mortality was 6.7% in the PPM group vs 4.7% in the other group (p=0.013).

The additive EuroSCORE was 6±3 in both groups (p=0.095). The logistic EuroSCORE was 8.64±9.29 in the group with PPM and 7.66±8.14 in the group without PPM (p=0.146).

### Predictors of early mortality

Univariate predictors of early mortality (shown in Table 3) included age, severely impaired left ventricular (LV) function (ejection fraction (EF) <30%), endocarditis, renal dysfunction, chronic obstructive pulmonary disease (COPD), cerebrovascular accident (CVA), concomitant coronary artery bypass grafting (CABG), CPB time, aortic cross-clamp time, previous cardiac surgery and PPM.

**Table 3:** Univariate and multivariate analysis of early (≤30 days) mortality

**Variable**		**Univariate analysis**		**Multivariate analysis**	
		***P***	**OR (95% CI)**	***P***	**OR (95% CI)**
Age, y		<0.001	1.04 (1.02-1.06)	<0.001	1.04 (1.02-1.07)
Sex (female)		0.058	1.42 (0.99-2.04)		
Severely impaired LV function	(EF <30%)	0.006	2.61 (1.32-5.14)	0.026	2.24 (1.10-4.56)
Hypertension		0.669	1.08 (0.75-1.56)		
Diabetes Mellitus		0.351	1.24 (0.79-1.94)		
Endocarditis		0.006	2.38 (1.28-4.44)	0.006	2.85 (1.36-5.96)
Body mass index (BMI), kg/m^2^		0.165	0.97 (0.93-1.01)		
Peripheral vascular disease (PVD)		0.052	1.65 (0.99-2.73)		
Renal dysfunction		<0.001	2.68 (1.54-4.64)	0.032	1.90 (1.06-3.40)
Chronic obstructive pulmonary disease (COPD)		0.005	1.78 (1.19-2.68)	0.029	1.61 (1.05-2.46)
Cerebrovascular accident (CVA)		0.013	2.20 (1.18-4.08)	0.110	1.71 (0.89-3.29)
Mechanical prosthetic valve		0.418	0.86 (0.60-1.24)		
Concomitant coronary artery bypass grafting (CABG)		0.004	1.71 (1.19-2.46)	0.872	0.96 (0.62-1.51)
Cardiopulmonary bypass (CPB) time, min		<0.001	1.02 (1.01-1.02)	<0.001	1.01 (1.01-1.02)
Aortic cross-clamp time, min		<0.001	1.02 (1.01-1.02)	0.691	1.00 (0.99-1.01)
Prosthesis-patient mismatch (PPM) (cut-off ≤ 0.85 cm^2^/m^2^)		0.014	1.80 (1.13-2.88)	0.120	1.50 (0.90-2.48)
Previous cardiac surgery		<0.001	2.96 (1.90-4.59)	0.072	1.62 (0.96-2.75)

*CI* = confidence interval; *EF* = ejection fraction; *OR* = Odds ratio; *LV* = left ventricular.

Multivariate predictors of early mortality did not include CVA, concomitant CABG, aortic cross-clamp time, previous cardiac surgery and the existence of PPM. Multivariate predictors of early mortality were age, severely impaired LV function, endocarditis, renal dysfunction, COPD and CPB time. This well-fitting model is showing non-significance on the Hosmer-Lemeshow goodness-of-fit test (p=0.308).

### Late mortality

Mean follow-up was 5.7±3.7 years (range 0.0-14.7 years). Total follow-up was 16728.6 patient-years. Late mortality for the entire group at 1, 5, and 10 years was 4%, 15% and 37%, respectively.

Late mortality at 1,5, and 10 years was 4%, 15% and 33%, respectively for the group without PPM and 4%, 16% and 43% for the PPM group.

### Predictors of late mortality

The results of Cox regression analysis for late mortality are shown in Table 4.

**Table 4:** Univariate and multivariate Cox analysis of late (>30 days) mortality

**Variable**		**Univariate analysis**		**Multivariate analysis**	
		***P***	**HR (95% CI)**	***P***	**HR (95% CI)**
Age, years		<0.001	1.07 (1.06-1.08)	<0.001	1.84 (1.33-2.56)
Sex (female)		0.403	1.07 (0.91-1.26)		
Severely impaired LV function	(EF <30%)	<0.001	1.93 (1.40-2.67)	<0.001	2.24 (1.10-4.56)
Hypertension		0.055	1.17 (0.99-1.37)		
Diabetes Mellitus		<0.001	1.80 (1.49-2.17)	0.001	1.41 (1.16-1.71)
Endocarditis		0.118	0.70 (0.45-1.09)		
Body mass index (BMI), kg/m^2^		0.837	1.00 (0.98-1.02)		
Peripheral vascular disease (PVD)		<0.001	2.61 (2.11-3.21)	<0.001	1.99 (1.61-2.46)
Renal dysfunction		<0.001	2.34 (1.76-3.13)	<0.001	1.69 (1.26-2.27)
Chronic obstructive pulmonary disease (COPD)		<0.001	1.64 (1.38-1.97)	<0.001	1.67 (1.39-1.99)
Cerebrovascular accident (CVA)		<0.001	1.88 (1.40-2.53)	<0.001	1.71 (1.27-2.31)
Mechanical prosthetic valve		<0.001	0.46 (0.39-0.54)	0.126	0.85 (0.70-1.05)
Concomitant coronary artery bypass grafting (CABG)		<0.001	1.58 (1.35-1.84)	0.993	0.99 (0.83-1.21)
Cardiopulmonary bypass (CPB) time, min		<0.001	1.01 (1.00-1.01)	0.001	1.01 (1.00-1.01)
Aortic cross-clamp time, min		<0.001	1.01 (1.00-1.01)	0.385	1.00 (0.99-1.00)
Prosthesis-patient mismatch (PPM) (cut-off ≤0.85 cm^2^/m^2^)		0.032	1.26 (1.02-1.55)	0.235	1.14 (0.92-1.42)
Previous cardiac surgery		<0.001	1.58 (1.25-2.00)	0.005	1.43 (1.11-.84)

CI = confidence interval; EF = ejection fraction; HR = hazard ratio; LV = left ventricular.

Univariate analysis revealed the following predictors of late mortality: age, severely impaired LV function, diabetes, peripheral vascular disease (PVD), renal dysfunction, COPD, history of CVA, the use of a mechanical prosthesis, concomitant CABG, CPB time, aortic cross-clamp time, previous cardiac surgery and PPM.

Multivariate analysis revealed the following independent predictors of late mortality: age, diabetes, severely impaired LV function, PVD, renal dysfunction, COPD, a history of CVA, CPB time and previous cardiac surgery. PPM was not an independent predictor of late mortality at multivariate analysis. Goodness of fit of the final model was assessed with the Chi-squared goodness-of-fit test: p<0.001.

### Long-term survival after AVR or AVR with CABG

**Figure 1:**
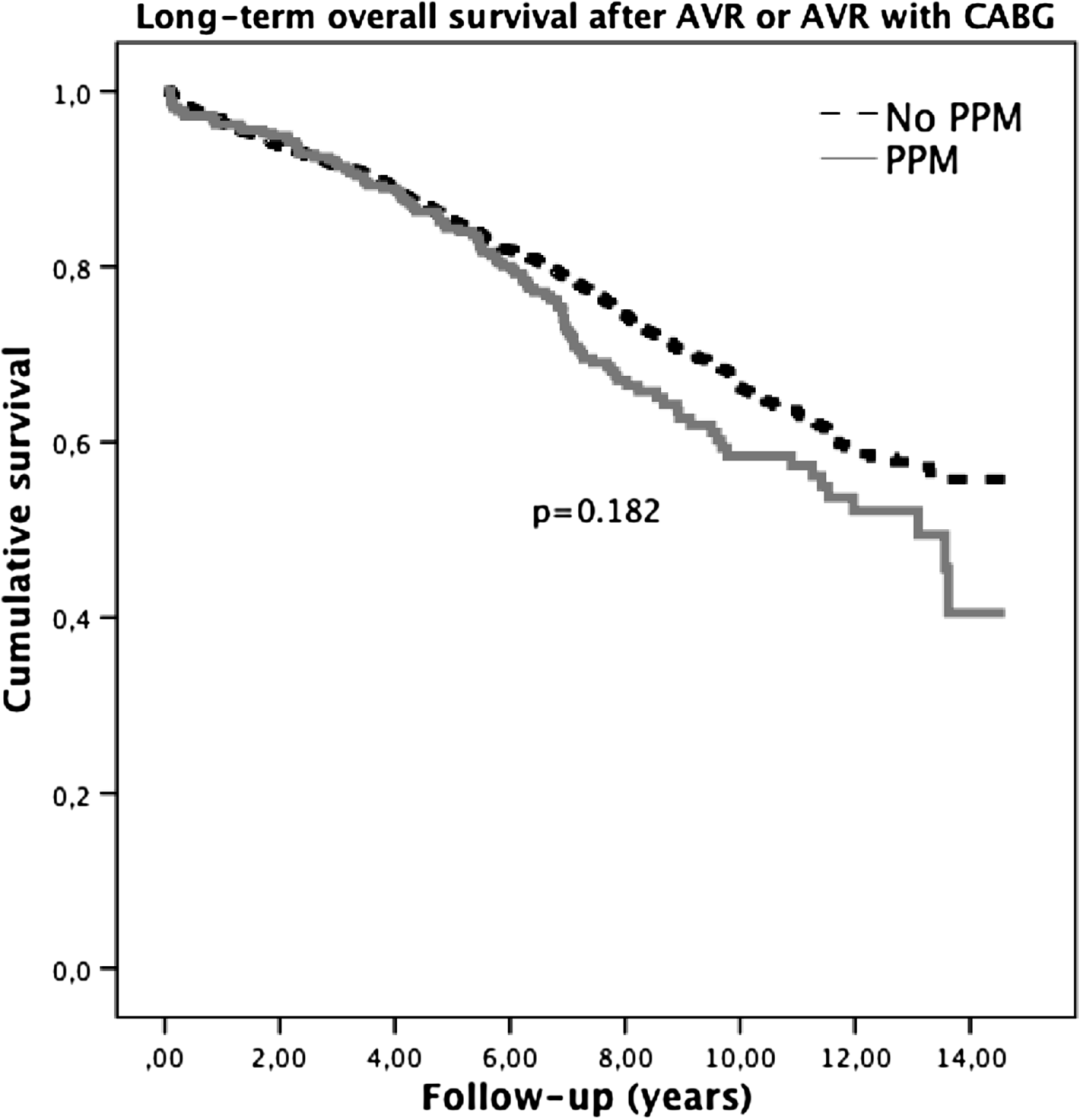
**Overall long-term survival after AVR and AVR with CABG stratified by PPM group.** The dotted line is showing long-term survival of the group without PPM and the full line is showing long-term survival of the group with PPM. There is no significant difference in survival between the two groups (p=0.182).

Figure 1 displays the long-term survival after AVR or AVR with CABG stratified by PPM group. Difference in survival between the groups by Breslow test was not significant (p=0.182).

## Discussion

The most important finding of this study is that PPM is not identified as an independent predictor of early or late mortality after AVR or AVR with CABG. Table 5 shows the independent predictors for early or late mortality after AVR or AVR with CABG. Diabetes, a history of CVA and PVD are not predictors of early mortality, but these comorbidities do have a detrimental impact on long-term survival. A history of previous cardiac surgery was an independent predictor of late mortality but not of early mortality, indicating that re-do cardiac surgery can be relatively safely performed with no significant negative effect on early survival. However most patients undergoing re-do cardiac surgery are in a more progressive phase of their disease which could explain the significant negative impact on long-term survival. Endocarditis was only a significant independent predictor of early mortality and did not have a negative effect on long-term survival. The risk-factors age, severely impaired LV function, renal dysfunction, COPD and CPB time were independent predictors of both early and late mortality.

**Table 5:** Multivariate independent predictors

**Variable**	**Early mortality**	**Late mortality**
Age	+	+
Severely impaired LV function (EF<30%)	+	+
Diabetes Mellitus	−	+
Endocarditis	+	−
Peripheral vascular disease (PVD)	−	+
Renal dysfunction	+	+
Chronic obstructive pulmonary disease (COPD)	+	+
Cerebrovascular accident (CVA)	−	+
Cardiopulmonary bypass (CPB) time	+	+
Previous cardiac surgery	−	+

+ = independent predictor of early and /or late mortality.

- = no independent predictor of early and/or late mortality.

The effect of PPM on early or long-term survival after AVR remains controversial [18]. Several studies have shown that PPM is a significant predictor of early or late mortality [3-6,16,19-21], whereas other studies did not report a significant association between PPM and early or late mortality [7-14,22-27]. These discrepancies may be caused by the different cut-off values for PPM (expressed in EOAI) and the different type of EOA values used to define PPM. Some authors use the projected indexed EOA derived from the published normal *in vivo* EOA values [2,5,17], whereas others use the *in vitro* manufacturer’s EOA [2,14,16]. We chose to use the *in vitro* EOA values provided by the manufacturers, since these values are uniform and publicly available for almost each type of prosthetic valve on the market. In our opinion, preoperative planning requires readily accessible and uniform information of all aortic valve prostheses currently available on the market. Manufacturer’s EOA charts are providing this information and most likely the surgeon would use these values to determine the possibility of PPM.

Valve prosthesis industry is a highly competitive market. Although most contemporary biological and mechanical prosthetic valve designs have excellent hemodynamic profiles, companies want to distinguish their products with demonstrable specifications. It would be illusory to deny the existence of PPM completely, but marketing may have caused too much attention concerning this topic. Perhaps the issue of PPM is partially an industry driven hype.

An important limitation is the retrospective design of this study. Although both study populations were homogeneous for most risk-factors, some baseline patient characteristics were significantly different between the two groups. We were not able to do subgroup analyses of moderate and severe PPM due to the lack of patients with severe PPM. This can be explained by our surgical strategy to implant the biggest valve possible and to use prosthetic valves with optimal hemodynamic profiles in patients with small annular sizes, thus inducing bias. On the other hand it illustrates that severe PPM is extremely rare when using this strategy even without using aortic annular enlargement techniques. Other studies confirm that the incidence of severe PPM is low [22,28]. In addition, the primary end-point was all-cause mortality. We were not able to retrieve the cause of death that might be equally important.

## Conclusions

In conclusion, our findings indicate that PPM is not an independent predictor for early or late mortality after AVR or AVR with CABG. The outcome of this study puts the importance of PPM once again into perspective.

## Abbreviations

AVR: Aortic valve replacement; BMI: Body mass index; BSA: Body surface area; CABG: Coronary artery bypass grafting; CI: Confidence interval; COPD: Chronic obstructive pulmonary disease; CPB: Cardiopulmonary bypass; CVA: Cerebrovascular accident; EF: Ejection fraction; EOA: Effective orifice area; EOAI: Effective orifice area index; HR: Hazard ratio; LV: Left ventricular; OR: Odds ratio; PPM: Prosthesis-patient mismatch; PVD: Peripheral vascular disease.

## Competing interests

All authors declare that they have no competing interests.

## Authors’ contributions

MS participated in the design of the study and helped to draft the manuscript. WB participated in the design of the study and helped to draft the manuscript. MM participated in the design of the study and revised the manuscript. KP made a substantial contribution to acquisition of data and interpretation of data. JD made a substantial contribution to acquisition of data and interpretation of data. AS made a substantial contribution to conception and design of the study, the acquisition and interpretation of data and revised the manuscript. BK made a substantial contribution to conception and design of the study, the interpretation of data, drafting the manuscript and performed the statistical analysis. All authors read and approved the final manuscript.
